# Identifying Significant SNPs of the Total Number of Piglets Born and Their Relationship with Leg Bumps in Pigs

**DOI:** 10.3390/biology13121034

**Published:** 2024-12-11

**Authors:** Siroj Bakoev, Lyubov Getmantseva, Maria Kolosova, Faridun Bakoev, Anatoly Kolosov, Elena Romanets, Varvara Shevtsova, Timofey Romanets, Yury Kolosov, Alexander Usatov

**Affiliations:** 1 Biotechnological Faculty, Don State Agrarian University, Persianovsky 346493, Russia; siroj1@yandex.ru (S.B.); m.leonovaa@mail.ru (M.K.); bakoevfaridun@yandex.ru (F.B.); lena9258@mail.ru (E.R.); timofey9258@mail.ru (T.R.); kolosov-dgau@mail.ru (Y.K.); 2 All Russian Research Institute of Animal Breeding, Lesnye Polyany 141212, Russia; kolosov777@gmail.com; 3 Southern Scientific Center Russian Academy of Sciences, Rostov-on-Don 344006, Russia; barbaragen4@mail.ru; 4 Academy of Biology and Biotechnology Named After D.I. Ivanovsky, Southern Federal University, Rostov-on-Don 344006, Russia; usatova@mail.ru

**Keywords:** pig, reproduction, total number of piglets born, bumps of legs, selection signature, GWAS, machine learning algorithm

## Abstract

The aim of this study was to identify genetic variants and pathways associated with the total number of piglets born and to investigate the potential negative consequences of the intensive selection for reproductive traits, particularly the formation of bumps on the legs of pigs. We used genome-wide association analysis and methods for identifying selection signatures. As a result, 47 SNPs were identified, localized in genes that play a significant role during sow pregnancy. These genes are involved in follicle growth and development (SGC), early embryonic development (CCDC3, LRRC8C, LRFN3, TNFRSF19), endometrial receptivity and implantation (NEBL), placentation, and embryonic development (ESRRG, GHRHR, TUSC3, NBAS). Several genes are associated with disorders of the nervous system and brain development (BCL11B, CDNF, ULK4, CC2D2A, KCNK2). Additionally, six SNPs are associated with the formation of bumps on the legs of pigs. These variants include intronic variants in the CCDC3, ULK4, and MINDY4 genes, as well as intergenic variants, regulatory region variants, and variants in the exons of non-coding transcripts. The results suggest important biological pathways and genetic variants associated with sow fertility and highlight the potential negative impacts on the health and physical condition of pigs.

## Simple Summary

To investigate the genetic architecture of the total number of piglets born, we applied two methods for evaluating genomic data: genome-wide association studies (GWAS) and the identification of selection signatures. We hypothesize that SNPs identified from GWAS and selection signatures can be considered as significant variants controlling reproductive processes associated with the total number born. Our results showed that significant SNPs are involved in key physiological processes during sow pregnancy. They are involved in follicular growth and development, early embryonic development, endometrial receptivity and implantation, placentation and embryonic development, and are associated with the risk of neurodevelopmental disorders and brain development. In addition, targeted selection for reproductive efficiency leads to changes in metabolic processes, which may be linked to the development of defects, in particular, leg bumps in pigs.

## 1. Introduction

The modern genetic techniques combined with appropriate methods of analysis can make a significant contribution to understanding the genetic architecture of complex traits. The term “complex” for phenotypes means the trait is influenced by several different genes (i.e., the trait is polygenic), as well as influenced by the environmental conditions [1]. Most indicators of farm animals’ productive traits relate to complex phenotypes.

One of the most important factors influencing the economic efficiency of pig farming is reproduction [2,3,4]. With the advent of new technologies and methods, the task of identifying loci associated with reproductive traits in pigs is increasingly attracting the attention of researchers. Improving reproductive efficiency, particularly the total number of piglets born (TNB), is of paramount importance in breeding efforts [5].

The number of piglets is a trait determined by a variety of regulatory gene pathways [6]. This trait is easy to measure and is fundamental for evaluating sows. However, in terms of physiological terms, TNB reflects a number of complex events that occur from the moment of fertilization to the birth of piglets and includes the formation of the zygote genome, implantation, embryonic development, etc. [7]. In this regard, a better understanding of the physiological components and the identification of the genes associated with these stages will contribute to improving and optimizing farm management strategies to maximize the reproductive success of sows.

In addition, the issue of health and welfare in farm animals is becoming more acute alongside the importance of their productivity [8]. Unfortunately, highly productive animals tend to experience a higher frequency of congenital defects and other health issues, and they are also less resistant to various diseases [9]. In pig farming, limb problems are one of the most common causes for sow cullings [10]. One type of limb problem is the appearance of bumps around the hock joints on the hind legs [11]. These defects are benign neoplasms of the connective tissue in hind legs and no pathogenic microflora has been observed in these neoplasms. In general, such defects do not lead to lameness but can affect the appearance of breeding pigs, making them unfit for sale. This, in turn, has a significant negative impact on the profitability of pig breeding centers. The results of studies [10,11] suggest that loci associated with tumor formation in the limbs are located in genes responsible for the metabolism of amino acids and fatty acids. It is also known that the metabolism of amino acids and fatty acids plays a crucial role in the early embryonic development of pigs [12,13,14]. In this context, it is important to understand whether targeted breeding efforts to increase reproductive performance could influence metabolic processes, potentially leading to the formation of bumps in highly productive purebred pigs.

To date, the Genome-wide Association Study (GWAS) is considered the “gold standard” for evaluating the genetic architecture of traits. After detecting associated SNPs, it is difficult to assess the nonlinear interactions between the SNPs and specific biological phenotypes [15]. Therefore, machine learning (ML) algorithms have clear advantages in searching for more complex patterns with higher prediction accuracy [16,17,18]. ML models have great potential for extracting patterns from single-nucleotide polymorphism datasets; however, the extent of their implementation in animal husbandry remains limited due to data limitations, complex genetic interactions, a lack of standardization, and reproducibility issues [19].

To identify genetic variants and biological pathways associated with certain phenotypic traits, ML models can be combined with GWAS and population genomics. Targeted artificial selection aimed at optimizing animal productivity has left marks in their genomes, known as selection signatures [20,21]. To identify loci that have undergone positive selection, various methods have been developed that scan genomes for such specific signals [22]. These signals can be based on haplotype homozygosity patterns [23,24]. Such methods include: iHS (integrated haplotype score), nSl (number of segregating sites by length), and iHH12 (integrated haplotype homozygosity pooled); these have become widely used in studies focused on identifying positive selection signals in humans and farm animals [25,26,27,28,29].

Selection signatures indicate genetic variants that have undergone selection and, therefore, point to loci with significant phenotypic variability. For this reason, they are of particular interest in mapping complex traits. The study of genetic variability shaped by selection provides valuable insights into the genetic mechanisms associated with productivity traits.

In this work, we aim to identify genetic variants and pathways associated with TNB and explore genetic associations that may contribute to the occurrence of defects, particularly bumps on the legs in pigs as a possible negative consequence of intense selection for reproductive traits.

## 2. Materials and Methods

The objectives of this study were: (1) to conduct a genome-wide association study for the phenotypic trait of total number born (TNB); (2) to compare the effect of various models and identify the most significant genetic variants as predictors for TNB; (3) to identify selection signatures in the studied population of Large White pigs; (4) to identify significant variants based on the assumption that these will be variants identified by both techniques—GWAS and selection signatures; (5) to determine the most significant physiological stages for the formation of the TNB phenotype based on the functional annotation of significant variants; and (6) to evaluate the effect of significant genetic variants for TNB on the predisposition to bump formation on the hock joints in pigs.

- **Animals and traits**

The studies were conducted on sows of the Large White breed (n = 1200). The Large White pigs used in this study were from a commercial pig breeding company in Russia and were imported from French lines. The population structure was determined using the PCA method with Plink 1.9 [30]. Detailed information about the study population was presented previously [31]. All animals were kept under the same feeding and housing conditions. Phenotype data for total number born (TNB) of piglets were collected from 2018 to 2023. The TNB data are presented in the Supplementary Figure S1. All sows had at least three parities. To account for the influence of additional factors (e.g., age differences, management differences if any were recorded, litter effects, farrowing effects, selection effects, preferential mating, etc.), phenotypes were adjusted into breeding value (EBV) estimates using BLUPF90 (1.01) [32].

To evaluate the association of SNPs with leg defects, pigs (n = 330) in which visual examination diagnosed the presence of bumps near the hock joints of the hind legs were selected. The effects of SNPs on the hock joints of the hind legs were assessed using the GCTA 1.94.1 program [33].

- **Genotyping**

Collecting ear samples is standard practice in swine production. The samples were genotyped using the GeneSeek^®^ GP SNP 80 × 1_XT (Illumina Inc., San Diego, CA, USA). The genotyping success rate was 0.999307. Genotype quality control and data filtering were performed using PLINK 1.9 [30]. The following quality control filters were applied: (geno 0.1, mind 0.1, maf 0.05). LD values were selected for the subsequent GWAS using the command (indep-pairwise 50 5 0.5).

- **GWAS/Models**

For GWAS, genotyping data and TNB phenotype data for Large White pigs (n = 1200) were used. GWAS for TNB was performed using five models, including four machine learning algorithms: Deep Learning (DL), Random Forest (RF), Gradient Boosting Machine (GBM), Extreme Gradient Boosting (XGBoost), and a linear Ridge Regression model (RR) with regularizers.

In contrast to traditional statistical methods, machine learning algorithms are better at capturing the complex relationships between genotypes and phenotypes.

The Random Forest (RF) algorithm was first proposed by Leo Breiman [34]. RF is an ensemble learning method that combines multiple models or algorithms to improve forecasting performance. The RF principle is based on generating multiple decision trees using m random samples selected via the bootstrap sampling method. In the case of a regression problem, the average forecast value of an individual tree is used as the model’s output. RF was chosen because it has a high degree of accuracy and is resistant to overfitting, even in the presence of a large number of redundant variables.

Gradient Boosting Machine (GBM) is a gradient boosting algorithm that combines several weak learning models to create a strong predictor. To calculate the difference between predicted and actual values, GBM uses a loss function and minimizes this loss using gradient descent when updating the model. XGBoost is an enhanced implementation of gradient boosting that improves performance by using a more regularized form of the model to control overfitting.

Deep Learning (DL), also known as a deep neural network, consists of multiple layers of connected neurons. Deep learning uses a multi-layer system of nonlinear filters to extract features with transformations, with each subsequent layer receiving the output of the previous layer as input.

The algorithms used provide a built-in feature importance ranking, which can be used to determine the most relevant SNPs. A threshold of the 0.9 quantile was applied, and only SNPs exceeding this threshold were selected.

Ridge Regression (RR) [35] compresses the coefficients of highly correlated variables (SNPs) relative to each other by adding a quadratic penalty on the magnitude of the coefficients to the problem of minimizing the sum of squared residuals. This process is also called l2-regularization or Tikhonov regularization. Penalties are introduced to avoid overfitting, reduce the variance of the prediction error, and handle correlated predictors. In RR, the main parameters are α, λ, and l2 (the sum of squared feature weights). Unlike Lasso regression, α is set to zero, and λ, the penalty parameter, is chosen using cross-validation (CV). The statistical significance of SNPs after multiple testing correction was tested using False Discovery Rate (FDR) with q < 0.01.

The models were built with the following scheme: correction/training, evaluation/confirmation, optimization of hyperparameters, and selection of significant predictors. All available data were divided into training and test sets in an 80%-20% ratio. The distribution was carried out in a random stratified manner. After splitting the dataset, missing values were imputed using Bagged Tree interpolation [36] or K-Nearest Neighbors [37] methods. Variables with near-zero variance were excluded. Standardization of numerical variables was performed using the max − min method, transforming the data into the range [0, 1]: Xnorm = (X − Xmin)/(Xmax − Xmin). The following metrics were used to evaluate and compare models: MAE, MSE, RMSE, R^2^, and RMSLE.

- **Selection signatures (SS)**

The key characteristic of positive selection is its ability to cause an unusually rapid increase in allele frequency, occurring in a short enough time that recombination does not significantly disrupt the haplotype on which the selected mutation occurs. Thus, a test for positive selection involves searching for a major haplotype with both high frequency and high extended haplotype homozygosity (EHH) relative to other major haplotypes at the locus. Based on homozygosity for the extended haplotype, several methods have been developed: iHS (integrated haplotype score), nSl (number of segregating sites by length), iHH12 (integrated haplotype homozygosity pooled), and others.

The Integrated Haplotype Score (iHS) is used to identify loci where strong selection has led to the emergence of new alleles and a decrease in haplotype homozygosity [23]. These alleles may be on the way to fixation or may become a balanced polymorphism. The Number of Segregating Sites by Length (nSl) is a statistic that serves a similar purpose to iHS, detecting both hard and soft selective sweeps. It is particularly robust to variations in recombination rates and the influence of demographic factors such as population subdivision, bottlenecks, and growth, and it does not require a genetic map [27]. Integrated Haplotype Homozygosity Pooled (iHH12), developed based on the H12 statistics [29], is able to scan for soft selective sweeps by adapting the H12 framework to iHH12 [28].

The iHS, nSl, and iHH12 approaches for detecting selection signals (SS) were implemented using the Selscan program 2.0 [38]. SNPs with a minor allele frequency (MAF) > 5% were retained for analysis, and each SNP was consistently treated as a major SNP. Unstandardized scores were normalized using the Selscan “norm” script. Windows with crit = 1 values were considered as candidate regions for selection [25].

- **Functional Annotation**

The genetic variants identified in the models that showed the best metrics were ranked by importance values. Variants that exceeded the 0.99 quantile were selected for further analysis. Gene search was performed in the Ensembl genome browser for Sscrofa 11.1. For all identified genes, enrichment analysis was performed using the ShinyGO v.0.81 database [39]. To search for quantitative trait loci (QTL), we used QTLdb (https://www.animalgenome.org/cgi-bin/QTLdb/SS/index, 8 December 2024) [40]. Enrichment analysis of the selection options with quantitative trait loci was carried out using the GALLO package for R program (https://www.r-project.org/) [41].

SNPs identified by GWAS and selection signatures were considered the most significant variants controlling reproductive processes and associated with the TNB phenotype. The most significant SNPs associated with TNB were then tested for a potential association with the predisposition to form bumps on the hock joints in pigs. A search for data on these significant SNP associations with traits in humans and animals was conducted through various open-access sources.

## 3. Results

### 3.1. Metrics of Models Deep Learning, Random Forest (RF), Gradient Boosting Machine (GBM), Extreme Gradient Boosting (Extreme Gradient Boosting (XGBoost)) and Ridge Regression (RR)

To achieve the highest accuracy in identifying significant genetic variants associated with the studied trait, five models were used, four of which were machine learning algorithms: Deep Learning, Random Forest (RF), Gradient Boosting Machine (GBM), Extreme Gradient Boosting (XGBoost), and a linear Ridge Regression model (RR).

To assess model quality, the following indicators were calculated: MAE, MSE, RMSE, R^2^, and RMSLE (Table 1). The quality of the models can be evaluated by comparing the indicator values from the training and test datasets.

**Figure 1:**
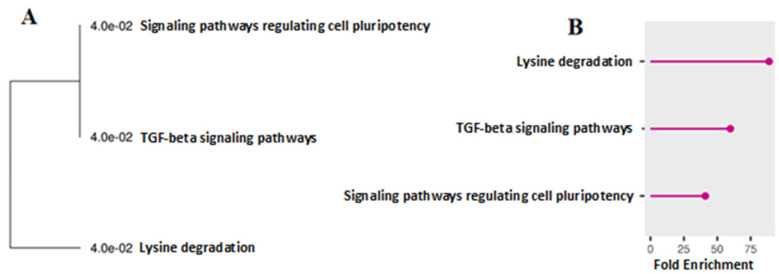
The results of enrichment analysis of the genes on the basis of the DL algorithm. Legend: (**A**) path tree; (**B**) distribution of paths by degree of enrichment.

Deep Learning (DL) and Ridge Regression (RR) outperformed the other models based on all evaluation criteria used in this study. The determination coefficients for DL and RR were 0.939 and 0.975, respectively. The genetic variants identified using the DL and RR models were ranked by importance, and those exceeding the 0.99 quantile were selected for further analysis. The results of the enrichment analysis of genes where significant genetic variants identified by the DL model were localized are shown in Figure 1. The main pathways identified include lysine degradation, TGF-beta signaling, and signaling pathways regulating cell pluripotency.

**Figure 2:**
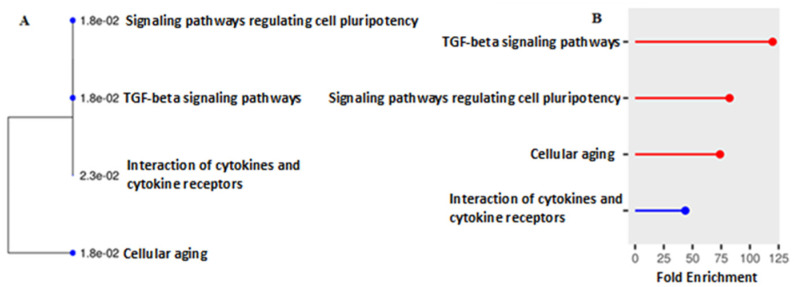
The results of enrichment analysis of the genes on the basis of the RR algorithm. Legend: (**A**) path tree; (**B**) distribution of paths by degree of enrichment.

The results of the enrichment of genes in which significant genetic variants identified by the RR model are localized are shown in Figure 2.

It should be noted that more than half of the significant genetic variants according to the DL and RR models were common (see Supplementary Table S1). Variants in the RR model are involved in key pathways of cellular senescence, cytokine and cytokine receptor interactions, and, similar to variants in the DL model, TGF-beta signaling pathways and signaling pathways regulating cellular pluripotency.

### 3.2. Selection Signatures

**Figure 3:**
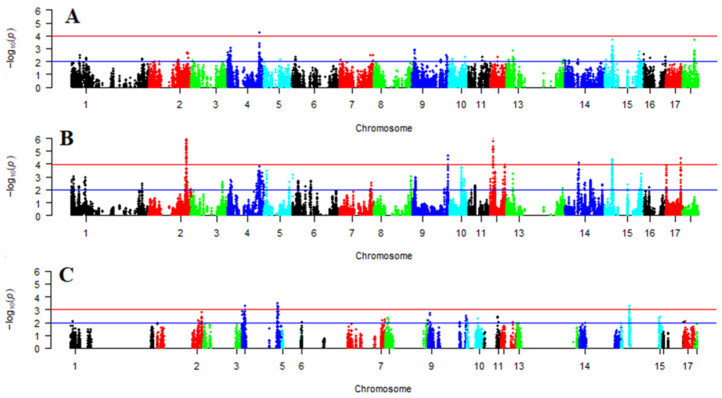
Distribution of selection signals. Legend: (**A**) distribution of iHS (integrated haplotype score) signals; (**B**) distribution of iHH12 (integrated haplotype homozygosity pooled) signals; (**C**) distribution of nSl (number of segregating sites by length) signals.

Since the frequency of the preferred allele is growing rapidly, the classical signal of rigid directional selection demonstrates a tendency to localize on an unusually long, slightly diverse haplotype. Based on the results of three methods, 2778 variants of selection signals (iHS—1124, nSl—563, and iHH12—1623) were determined (see Supplementary Table S2). The distribution of selection signals in pigs is shown in Figure 3.

**Figure 4:**
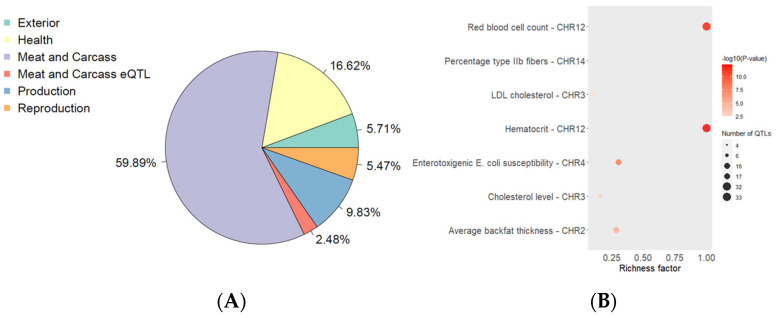
Enrichment of selection signals with quantitative trait loci. Legend: *(***A**) enrichment at the QTLs type; *(***B**) enrichment at the feature level.

A total of 808 genes were found to be under selection pressure. The localization of genetic selection variants revealed genes that intersect with loci responsible for meat and carcass traits in pigs. Selection also affected loci related to health, productivity, reproduction, and exterior traits (Figure 4).

### 3.3. Significant Variants Identified by Both GWAS and Selection Signature Methods

In addition, common variants identified by DL and RR models and selection signatures detected earlier by iHS, nSL, and iHH12 methods in this population were identified (Table 2).

### 3.4. Functional Annotation of Signification Variants and Their Probable Role in the Formation of the TNB Phenotype

These variants are localized in the genes NBAS, LRRC8C, LRFN3, BCL11B, CC2D2A, KCNK2, ESRRG, ENSSSCG00000039658, NEBL, CCDC3, TNFRSF19, FAM117A, ULK4, SGCZ, TUSC3, GHRHR, and MINDY4. All these genes are more or less associated with complex processes accompanying the gestation of sows and the continuous changes that occur in both the yellow bodies and the endometrium, depending on the embryonic, preimplantation, or intrauterine stages.

The functional unit of the mammalian ovary is the follicle. As follicles develop, somatic cells proliferate and differentiate. The granulosa cells lining the oocyte become capable of responding to sex steroids, including estrogens, androgens, and progestogens [42]. Folliculogenesis is a complex process that requires well-regulated gene expression and interactions [43]. MicroRNAs (miRNAs) play a decisive role in ovarian follicle development by influencing genes involved in folliculogenesis [44]. For example, miRNA-383 may participate in granulosa cell proliferation and the development and maturation of oocytes [45]. Transcription of miRNA-383 is controlled by the Zeta-Sarcoglycan gene (SGCZ), which was previously identified by the DL and RR models as a marker of pig fertility and also underwent positive selection in this population. MicroRNA-383 is located in the first intron of the SGCZ gene. Intron microRNAs can originate from a common transcript with host genes or be independently transcribed using their own promoter, similar to RNA polymerase II [46].

In the work of Gu et al. [45], it was shown that miRNA-383 is co-expressed with the SGCZ gene in mouse tissues and in granulosa cells treated with TGF-β1, indicating that they are co-transcribed. Based on this, it can be assumed that the SGCZ gene plays an important role in regulating ovarian follicle growth and fertility in pigs as well. In addition, this gene has undergone positive selection in the studied population.

Early mammalian embryonic development consists of a series of highly conserved, regulated, and predictable cell division events, including fertilization, fragmentation, activation of the zygotic genome, morula densification, and blastocyst formation. In the early stages of embryonic development, control is carried out by maternal transcripts and proteins. Subsequently, activation of the zygotic genome is initiated, and maternal factors gradually disappear. Activation of the zygotic genome is a critical process in early embryonic development [47]. Any disruption of these processes can negatively affect embryonic development.

In pigs, zygotic genome activation occurs from the 2-cell to the 4-cell stage of embryo development [48]. On the fourth day after fertilization, the embryo undergoes a compaction process, which is associated with increased intercellular adhesion and the acquisition of cell polarity, giving it a mulberry-like appearance, called a morula. Blastocyst formation occurs on the fifth day. This process is closely related to the inner cell mass (ICM) and the specification of the trophectoderm (TE).

One of the genes under positive selection found in the DL and RR models is the Coiled-Coil Domain-Containing Protein 3 (CCDC3). This gene encodes a protein expressed in endothelial cells and adipose tissue and it positively regulates adipogenesis and lipid accumulation. Cao et al. [12] analyzed the transcriptomes of preimplantation pig and mouse embryos. CCDC3 gene expression was observed in both pigs and mice, and the authors concluded that trophectoderm differentiation in embryos of these species is regulated by different signaling pathways. This suggests that CCDC3 plays a significant role in preimplantation embryonic development, including in pigs.

The work by Aikawa et al. [49] presents the results of studying gene expression in mice during early pregnancy. The researchers found that gene expression levels in epithelial cells change dynamically over time, which may be related to different transcription mechanisms at specific time intervals. Thus, it is hypothesized that, on day 5, gene expression—specifically of Leucine-Rich Repeat-Containing Protein 8C (LRRC8C) and Leucine-Rich Repeat And Fibronectin Type-III Domain-Containing Protein 3 (LRFN3)—increases in the epithelium, angiogenesis is activated, and cytokine pathways are triggered. The permeability of blood vessels surrounding the implantation sites increases. Importantly, the LRRC8C gene encodes one subunit of a heteromeric protein that regulates the volume of anion channels. LRR domains are evolutionarily conserved protein structures, and researchers hypothesize that they contribute to an innate immune response. The role of LRRC8C has been reported in many immune processes [50]. In the work of de Castro et al. [51], LRRC8C is considered one of the genes involved in embryo-endothelium interaction in mares. The endometrium is crucial for blastocyst implantation, and any disruptions in this process jeopardize subsequent pregnancy outcomes.

Various cytokine genes are associated with lipid metabolism in pigs. The Tumor Necrosis Factor Receptor Superfamily Member 19 (TNFRSF19) gene is a member of the tumor necrosis factor receptor superfamily and is involved in the interaction between cytokines and cytokine receptors [52]. In pigs, the TNFRSF19 gene may play a crucial role in lipid accumulation and be relevant to fatty acid metabolism. Mekchay et al. [53] showed that variants in the TNFRSF19 gene are associated with intramuscular fat content and arachidonic acid levels in pigs. In addition, high expression of TNFRSF19 is observed during embryo development. All these factors may indicate a significant role of the TNFRSF19 gene in embryonic development and fertility in pigs.

A number of genes identified by DL and RR analyses, along with selection signatures, are associated with processes that ensure endometrial receptivity and embryo implantation. Endometrial receptivity is achieved through significant structural changes in the cytoskeleton and plasma membrane of epithelial cells, which, in turn, facilitate embryo adhesion. For example, the Nebulette gene (NEBL) encodes a protein belonging to the nebulin family of actin-binding proteins that are important in myofibrillogenesis and Z-line assembly. Some scientists hypothesize that variants in the NEBL gene are associated with various cardiomyopathies [54]. In addition to cardiologic pathologies, reduced NEBL expression may lead to poor epithelial receptivity and, as a result, failed embryo implantation. Zhou et al. [55] determined that in women with recurrent implantation failure, Actinin Alpha 1 (ACTN1) levels were significantly increased, while NEBL levels were decreased. Overexpression of ACTN1 significantly reduced NEBL levels, increasing F-actin fibers and impairing blastocyst adhesion, thus mimicking the process of impaired embryo adhesion. Conversely, overexpression of NEBL markedly restored adhesion.

The most significant embryo loss in pigs occurs during implantation in early gestation, with mortality reaching 30% between ten and thirty days of gestation [56]. Uteroplacental insufficiency is believed to be the main cause. The placenta is essential for fetal development and the successful outcome of pregnancy, serving as an organ for nutrition, protection, gas exchange, and regulation of fetal growth and development. Any changes in the structure of the placenta may be associated with the risk of neurodevelopmental disorders, leading to delayed fetal development and the development of various anomalies. A number of genes identified from the DL and RR analyses and selection signatures can be considered in this context.

The Radiation-Induced Tumor Suppressor Gene 1 Protein (BCL11B) encodes a transcription factor required for postnatal hippocampal development. Deficiency of this factor can lead to structural defects in the brain [57]. In addition, BCL11B is involved in T-cell lineage determination and maintenance, mediating the development of innate lymphoid cell group 2 (ILC2) cells that produce type 2 effector cytokines [58]. In humans, genetic variants in the BCL11B gene are associated with impaired T-cell development [59] and delayed intrauterine development. It is also known that insufficient expression of BCL11B can lead to immune and neuronal imbalances [60].

The glial cell lineage-derived neurotrophic factor (CDNF) is a member of the TGF-β superfamily of growth factors with neurotrophic activity against midbrain dopaminergic neurons. GDNF-null mice, regardless of their target mutation, demonstrate complete agenesis of the kidneys due to lack of induction of the ureter rudiment. These mice also lack enteric neurons [61].

The Serine/Threonine-Protein Kinase (ULK4) gene encodes a protein that performs several functions in the brain, including participation in neuronal cell proliferation and cell cycle regulation. Functions outside the central nervous system remain largely unknown [62].

The Coiled-Coil and C2 Domain-Containing 2A (CC2D2A) gene encodes a protein that plays a crucial role in cilia formation. Primary cilia participate in many different signaling pathways, but the molecular mechanisms underlying the sensory function of primary cilia are poorly understood. Cilia dysfunction underlies the development of severe diseases commonly referred to as ciliopathies [63]. Ciliopathies have a pleiotropic phenotype and affect multiple organs. Tsyklauri et al. [64] presented an association between ciliopathies and dysregulation of the immune and hematopoietic systems. Typical symptoms of ciliopathies may occur due to cilia dysfunction during embryonic development. Genetic variants in CC2D2A are associated with a spectrum of different ciliopathy syndromes, including neurological diseases such as Joubert syndrome and Meckel syndrome [65]. While how these variants lead to such a wide spectrum of clinical manifestations with partially overlapping phenotypes is not fully understood, the results obtained earlier from selection models and signatures suggest that the CC2D2A gene plays a significant role in sow fertility.

Precise control of ionic flux (calcium, sodium, and potassium) contributes to intrauterine developmental processes, such as the proliferation, migration, and differentiation of neurons [63]. Ion channel dysfunction causes a number of pathologies known as channelopathies. The Potassium Two-Pore Domain Channel Subfamily K Member 2 (KCNK2) gene, also known as TREK1, encodes a protein belonging to the potassium channel family with a two-pore domain. High expression of KCNK2 has been reported in several brain regions, including the hippocampus, cerebellum, olfactory medulla, and cerebral cortex [66]. Due to its localization, this gene has been considered a target for a number of pathologies affecting the central nervous system. Ion channel dysfunction has traditionally been studied in the context of postnatal differentiated neurons. However, its role in prenatal brain abnormalities, which can lead to various cortical malformations and developmental issues, is now recognized [67]. Although the role of this gene in embryonic development and fecundity in pigs remains unknown, its involvement in physiological and pathophysiological processes and its appearance in models searching for associations with fecundity and selection signatures make it a promising target for further research in the context of pig reproduction.

The Tumor Suppressor Candidate 3 (TUSC3) gene is required for cellular magnesium uptake, which in turn plays a critical role in embryonic development [68]. TUSC3 expression is highest in the ovaries, placenta, prostate, testes, adipose tissue, and lungs [69]. Zhou et al. demonstrated that the MagT1 and TUSC3 genes are essential for cellular Mg^2^⁺ transport and vertebrate embryonic development [70]. These genes play a central role in vertebrate embryonic development, a function that cannot be compensated by other magnesium transporters. In pigs, magnesium plays an important role in reproductive traits such as the wean-to-estrus interval and total number of piglets born. Magnesium also affects the digestibility of crude fiber and crude protein during late gestation and lactation.

The significant genetic variants were localized in the following genes: NBAS, LRRC8C, LRFN3, BCL11B, CC2D2A, KCNK2, ESRRG, GDNF, NEBL, CCDC3, TNFRSF19, FAM117A, ULK4, SGCZ, TUSC3, GHRHR, MINDY4, and INMT. These genes play significant roles during sow gestation and are involved in various processes such as follicle growth and development (SGCZ), early embryonic development (CCDC3, LRRC8C, LRFN3, TNFRSF19), endometrial receptivity and implantation (NEBL), placentation, and embryonic development (ESRRG, GHRHR, TUSC3, NBAS). It is also important to note that several of these genes are associated with the risk of impaired development of the nervous system and brain, which can subsequently provoke delayed embryonic development and lead to anomalies (BCL11B, CDNF, ULK4, CC2D2A, KCNK2).

### 3.5. Effect of Signification Genetic Variants for TNB on the Predisposition to Bumps Formation on the Hock Joints in Pigs

Limb defects are a complex trait that requires comprehensive study. In this work, for the first time, the most significant variants associated with the total number of piglets born (TNB) were tested for a potential association with a predisposition to the formation of bumps on the hock joints of the hind legs in pigs. As a result, six SNPs were identified that showed a strong statistical relationship with the bumps phenotype (Table 3).

The SNPs associated with the studied trait are intron variants in the CCDC3, ULK4, and MINDY4 genes, as well as intergenic variants, regulatory region variants, and non-coding transcript exon variants. Although their functions have not been fully studied, it can be inferred that these variants play a role in the physiological processes related to the formation of bumps in pigs.

The CCDC3 gene is widely expressed in the skin and adipose tissue and is involved in the negative regulation of gene expression, a signaling pathway mediated by the tumor necrosis factor, and the dysregulation of lipid metabolism [71].

The ULK4 gene plays a key role in neurogenesis and corticogenesis during various developmental stages. In commercial pig hybrids, this gene is associated with growth parameters [72]. It is also thought that ULK4 may contribute to ciliopathies [73].

The MINDY4 gene, also known as MINDY Lysine 48 Deubiquitinase 4, is implicated in the development of pathological conditions such as cancer and neurological disorders.

## 4. Conclusions

In summary, the GWAS results and identification of selection signatures revealed significant variants that play a critical role in determining the total number of piglets born. These SNPs are located in genes involved in key aspects of sow pregnancy, including follicular growth, embryonic development, endometrial receptivity, and placentation. Notably, some of these genes are associated with neurodevelopmental disorders, which could delay embryonic development and lead to abnormalities. Additionally, several SNPs are linked to susceptibility to bump formation in pigs. The results underscore the potential of these genetic variants as fertility markers and highlight their possible impact on the health and physical performance of pigs, suggesting avenues for further research in reproductive biology and disease prevention.

## Figures and Tables

**Table 1:** Model metrics.

Metrics	Training Dataset		Test Datasets
	Mean Value	Standard Deviation	Mean Value
Deep Learning (DL)			
Mae	0.590020	0.071773	0.4750844
mean_residual_deviance	0.559832	0.128388	0.3502782
Mse	0.559832	0.128388	0.3502782
r^2^	0.939155	0.011273	0.9618232
residual_deviance	0.559832	0.128388	0.3502782
Rmse	0.745025	0.084581	0.5918431
Rmsle	0.043911	0.005592	0.03451773
Extremal Gradient Boosting (XGBoost)			
Mae	1.510068	0.071681	1.871898
mean_residual_deviance	3.617699	0.413383	5.514274
Mse	3.617699	0.413383	5.514274
r^2^	0.604665	0.031571	0.3989991
residual_deviance	3.617699	0.413383	5.514274
Rmse	1.899250	0.108266	2.348249
Rmsle	0.111984	0.008008	0.1389622
Gradient Boosting Machine (GBM)			
Mae	1.884282	0.075253	1.863851
mean_residual_deviance	5.517185	0.427668	5.470074
Mse	5.517185	0.427668	5.470074
r^2^	0.393505	0.050699	0.4038164
residual_deviance	5.517185	0.427668	5.470074
Rmse	2.347314	0.090063	2.338819
Rmsle	0.137453	0.005693	0.1384256
Random Forest (RF)			
Mae	1.983754	0.106916	0.7669743
mean_residual_deviance	5.979893	0.606600	0.9717731
Mse	5.979893	0.606600	0.9717731
r^2^	0.343705	0.041655	0.3938947
residual_deviance	5.979893	0.606600	0.9717731
Rmse	2.442547	0.124092	0.9857855
Rmsle	0.143224	0.008028	0.05970351
Ridge Regression (RR)			
Mae	0.383172	0.017936	0.7669743
mean_residual_deviance	0.233283	0.031785	0.9717731
Mse	0.233283	0.031785	0.9717731
null_deviance	2756.310800	249.617690	0.8938947
r^2^	0.974526	0.002510	0.9717731
residual_deviance	70.136820	10.107722	0.9857855
Rmse	0.482020	0.032305	0.05970351

**Table 2:** Genetic variants identified by DL and RR models and selection signatures.

SSC	Position	iHH12	iHS	nSL	RR	DL	rs	Type of Variants	Gene
1	26068717	2.10	-	-	0.53	-	rs80949671	upstream gene variant	*LncRNA*
1	26505944	2.16	-	-	0.51	-	rs81351637	intergenic variant	
1	26593023	-	2.56	2.14	0.52	-	rs81351644	intergenic variant	
1	26625743	-	2.47	2.04	0.61	0.67	rs81351664	intergenic variant	
1	30564821	-	2.46	-	0.75	-	rs80857633	intergenic variant	
3	122158369	-	2.05	-	0.51	-	rs81377655	intron variant	*NBAS*
4	125690120	-	2.22	2.30	0.50	-	rs81339593	intergenic variant	
4	126987143	-	-	2.01	0.58	0.67	rs80958447	intron variant	*LRRC8C*
5	10852290	-	2.17	-	-	0.69	rs80842148	intergenic variant	
6	45342655	2.52	-	-	0.64	-	rs81395771	upstream gene variant	*LRFN3*
7	14152430	-	2.15	2.04	0.52	-	rs326183563	intron variant	*LncRNA*
7	108105220	2.18	-	-	0.50	-	rs80793422	intergenic variant	
7	120229737	-	2.66	-	0.50	-	rs80808822	intron variant	*BCL11B*
7	120610621	-	2.96	-	0.56	0.66	rs80935654	intergenic variant	
8	10796231	-	-	2.31	-	0.71	rs334772761	intron variant	*CC2D2A*
9	128673870	2.18	2.28	-	0.53	0.65	rs335981516	intergenic variant	*KCNK2*
10	6950011	-	2.14	-	-	0.67	rs81428487	intergenic variant	*ESRRG*
10	47212377	-	2.11	-	0.58	-	rs81477650	intron variant	*CDNF*
10	53675235	2.43	-	-	0.61	-	rs339993261	intron variant	*NEBL*
10	53697286	2.28	-	-	0.51	0.63	rs81314157	intron variant	*NEBL*
10	53738869	2.21	-	-	0.57	0.77	rs81241974	intron variant	*NEBL*
10	59106264	-	2.29	2.09	0.51	-	rs81426512	intron variant	*CCDC3*
11	2538185	-	2.22	-	-	0.67	rs331680910	intergenic variant	*TNFRSF19*
12	25912338	-	2.86	-	0.53	-	rs81239149	intron variant	*FAM117A*
13	25296621	2.45	-	-	-	0.65	rs81284602	intron variant	*ULK4*
14	123823825	-	2.48	-	0.59	0.71	rs320763504	intergenic variant	
15	29638798	2.05	2.45	2.60	0.70	0.84	rs81452296	intergenic variant	
15	121460992	-	-	2.08	0.60	-	rs341236717	intron variant	*ENSSSCG00000032522*
15	121927668	-	3.16	2.92	0.51	-	rs81455316	intergenic variant	
15	121959926	2.07	2.38	2.26	0.62	-	rs81455304	regulatory region variant	
15	122065360	2.37	-	-	0.59	0.69	rs325379930	upstream gene variant	
15	122153233	2.19	2.77	2.48	0.59	0.67	rs337997805	intergenic variant	
15	122263611	2.39	-	-	0.56	-	rs80856455	intergenic variant	
15	122294134	2.27	-	-	0.69	0.72	rs80965619	intergenic variant	
15	122374213	2.06	2.34	2.33	0.59	-	rs318986640	intergenic variant	
16	7850860	-	2.07	-	0.56	-	rs81345229	intergenic variant	
17	2890084	2.22	-	-	0.50	-	rs81465731	intron variant	
17	3013297	2.12	-	-	0.55	-	rs81465735	intron variant	*SGCZ*
17	3316742	2.61	-	-	-	0.69	rs81466107	intron variant	*TUSC3*
17	3448507	2.00	-	-	-	0.69	rs3471300045	intron variant	*TUSC3*
17	40763707	-	2.04	-	0.52	-	rs318485305	intergenic variant	
17	53760514	3.76	-	-	0.55	-	rs80892682	intergenic variant	
17	53970256	2.39	-	-	0.56	-			
18	41692890	2.04	2.25	-	0.51	0.73	rs319528527	intergenic variant	
18	41992583	-	2.61	-	0.52	-	rs81339141	regulatory region variant	
18	42038739	-	3.14	-	0.54	-	rs337936594	intron variant	*GHRHR*
18	42078871	-	3.20	-	0.51	-			
18	42126037	-	3.70	-	0.54	-	rs80945004	intron variant	*MINDY4*
18	42222435	-	2.93	-	0.63	0.69	rs324752471	downstream gene variant	*INMT*
18	42547236	-	2.25	-	0.67	0.90	rs341575491	non-coding transcript exon variant	

Legend: SSC (Sus Scrofa chromosome), iHH12 (integrated haplotype homozygosity pooled), iHS (integrated haplotype score), nSl (number of segregating sites by length), DL (Deep Learning), RR (Ridge Regression).

**Table 3:** SNPs associated with phenotype bumps.

Chrom	Position	rs	Type of Variant	Gene	*p*-Value
10	59106264	rs81426512	intron variant	*CCDC3*	0.00010950
13	25296621	rs81284602	intron variant	*ULK4*	0.00015930
18	41692890	rs319528527	intergenic variant	*NEUROD6*	0.00000830
18	41992583	rs81339141	regulatory region variant	*ADCYAP1R1*	0.00000030
18	42126037	rs80945004	intron variant	*MINDY4*	0.00000011
18	42547236	rs341575491	non-coding transcript exon variant	*ZNRF2*	0.00000007

## Data Availability

Data is available upon reasonable request.

## Supplementary Materials

The following supporting information can be downloaded at: https://www.mdpi.com/article/10.3390/biology13121034/s1, Figure S1: Distribution of data for total number born of piglets; Table S1: Significant genetic variants according to the DL and RR models; Table S2: Selection signals according to the iHS, nSl, and iHH12 methods.
