# Supplementary material for: Identifying Significant SNPs of the Total Number of Piglets Born and Their Relationship with Leg Bumps in Pigs
Source: Biology (Basel). 2024 Dec 11;13(12):1034. doi: 10.3390/biology13121034 (PMC11673605; doi:10.3390/biology13121034)
Supplement: Supplementary file 1 [file biology-13-01034-s001.zip › Figure S1.pdf]

# Identifying Significant SNPs of the Total Number of Piglets Born and Their Relationship with Leg Bumps in Pigs

**Siroj Bakoev [1], Lyubov Getmantseva [1,*], Maria Kolosova [1], Faridun Bakoev [1], Anatoly Kolosov [2], Elena Romanets [1], Varvara Shevtsova [3], Timofey Romanets [1], Yury Kolosov [1] and Alexander Usatov [4]**

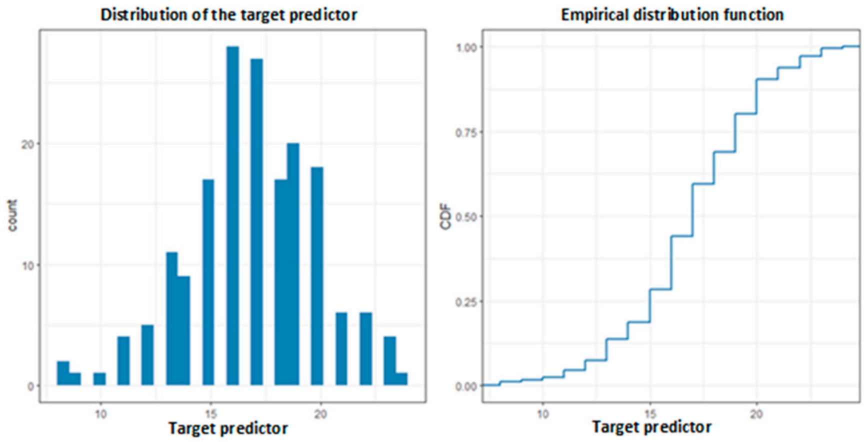


Figure S1. Distribution of data for total number born of piglets.
